# BEAP: The BLAST Extension and Alignment Program- a tool for contig construction and analysis of preliminary genome sequence

**DOI:** 10.1186/1756-0500-2-11

**Published:** 2009-01-22

**Authors:** James E Koltes, Zhi-Liang Hu, Eric Fritz, James M Reecy

**Affiliations:** 1 Department of Animal Science, Iowa State University, Ames, Iowa 50011-3150, USA

## Abstract

**Background:**

Fine-mapping projects require a high density of SNP markers and positional candidate gene sequences. In species with incomplete genomic sequence, the DNA sequences needed to generate markers for fine-mapping within a linkage analysis confidence interval may be available but may not have been assembled. To manually piece these sequences together is laborious and costly. Moreover, annotation and assembly of short, incomplete DNA sequences is time consuming and not always straightforward.

**Findings:**

We have created a tool called BEAP that combines BLAST and CAP3 to retrieve sequences and construct contigs for localized genomic regions in species with unfinished sequence drafts. The rational is that a completed genome can be used as a template to query target genomic sequence for closing the gaps or extending contig sequence length in species whose genome is incomplete on the basis that good homology exists. Each user must define what template sequence is appropriate based on comparative mapping data such as radiation hybrid (RH) maps or other evidence linking the gene sequence of the template species to the target species.

**Conclusion:**

The BEAP software creates contigs suitable for discovery of orthologous genes for positional cloning. The resulting sequence alignments can be viewed graphically with a Java graphical user interface (GUI), allowing users to evaluate contig sequence quality and predict SNPs. We demonstrate the successful use of BEAP to generate genomic template sequence for positional cloning of the Angus dwarfism mutation. The software is available for free online for use on UNIX systems at .

## Methods

### BEAP construction

The BLAST and CAP3 processes were linked via Perl scripts to create a sequence assembly pipeline (see Additional file 1 for rationale, Additional File 1 Figure S5 and Additional file 2) BLAST was looped in an iterative process such that after all the queries, each unique sequence was used as template for the next round of BLAST against the initial databases. After all sequences were retrieved, they were sent to CAP3 for assembly using the default settings and available sequence quality files from NCBI. The CAP3 output, saved as a text file, can then be manually uploaded using the BEAP GUI. A detailed discussion of results from testing of BEAP options is presented in Additional File 1.

Several internal features were created to enhance and monitor BEAP performance. A filter was created to remove sequences retrieved multiple times to limit the number of frivolously repeated BLAST queries. The number of BLAST rounds was also monitored such that only the desired number of reiterative BLAST rounds was performed, up to a maximum number defined by the user. This method allowed BEAP to stop the re-iterative BLAST process if no new sequences were retrieved, reducing the amount of time needed to complete a BEAP assembly. A progress summary was also kept in the log file throughout the process to keep track of sequences retrieved and corresponding statistics such as sequence ID, database or origin, number of bases, E-value, etc.

### Development of the BEAP GUI for sequence alignment visualization

The BEAP GUI was built as extension of BEAP to improve assembled sequence analysis. The GUI allows a more in depth examination of individual sequences and contigs in more viewer friendly environment for those not used to raw program output. The GUI consists of a main window that is opened upon execution of the program. Features include a button for contig file uploads, a selection box to change contig views and a viewer panel in which the contig and all the sequences used to create it are visualized.

Visualization of contigs and their accompanying sequence members was accomplished by parsing through the output files generated by CAP3 using the designations for sequence IDs from NCBI, ie. CO, BQ, AF, RD, and QA. The program uses the parsed information from the CAP3 file to create a contig object that has associated an array of sequence objects composed of the sequence that was used to create this contig. The contig object itself is then put into an array. The program then passes the array of contig objects to the other parts of the program responsible for creating views, saving images of these views, and printing of the views.

### Software development

BEAP is written in Perl 5.8.0 and tested on a Red Hat Linux Application Server 3.0. The NCBI BLAST (version 2.2.9) and CAP3 (version date: 08/29/2002) software are utilized by BEAP to serve the purpose of sequence searching and assembling. Currently, BEAP is a command line program run on a Linux terminal. Planning is under way that it will be implemented as a web interfaced program for public access. The GUI tool was developed with Java (version 1.5.X) and tested on Windows XP and Mac OS X.4.11. The BEAP program was developed and tested on a 533 MHz dual processor Linux computer with 8 GB RAM.

### BEAP performance testing

Since many options were inherently flexible in BEAP, we wanted to test a number of scenarios that could alter BEAP performance. To determine how BEAP output was changed when BLAST settings were altered, we varied the E-value stringency, word size and number of databases. To determine how different sequence attributes would affect BEAP performance, we compared output generated when using intronic, exonic, exonic and untranslated regions (UTR), and varying levels of repetitive sequence elements template sequence. To determine how template sequence size altered BEAP performance, we tested individual template sequence sizes, the number of sequences used as template, and the total amount of sequence used as template. Last, we investigated the difference in BEAP output when using local (megaBLAST) vs. network BLAST query.

### Template sequence

The user must define the appropriate template sequence and species. The template sequence is used much like primers in PCR for BLAST to query the species of interest. Cross-species comparative maps (i.e. RH maps) can be used to identify syntenic sequence blocks between species to find a suitable template sequence.

### Application: the use of BEAP to construct contigs within the Angus dwarfism locus

Fine-mapping of the Angus dwarfism locus resulted in a critical region of roughly 1–2 Mbps on *Bos taurus *autosome (BTA) 6. Since the bovine genome was not fully sequenced upon the first application of BEAP in 2005, many of the candidate genes in this genomic region were unknown and unannotated. The Human-Bovine RH map was used to define the template sequence allowing for some extra sequence proximal and distal to the homologous bovine chromosome block. *Homo sapien *autosome (HSA) 4 genomic DNA sequence from 78,000,000 to 83,000,000 base pairs was defined as the "template" for BEAP assembly of the bovine. This genomic block contained 20 genes and pseudo-genes [1,2]. The template sequence used by BEAP included both exonic and UTR sequences. All template sequences were retrieved from UCSC genome browser [3] and the ENSEMBL database [4]. Repetitive sequences were masked using Repeat Masker software in the template (i.e. human sequence) prior to use of BEAP [5]. We used RH markers within genes in both the bovine and human genome builds to anchor the template to the target physical map. An E-value of e-30 was used for all tests. We queried sequence from six NCBI databases (see Additional File 1). The sequences obtained in the application to the bovine dwarfism locus used the sequence databases available in 2005, prior to full bovine genome assembly. The BEAP performance trials utilized the whole bovine genome sequence, version 3, from 2007.

## Results

### Performance of BEAP

BEAP test results indicated contigs spanning exons and flanking intronic sequences, allowing researchers to find gene sequences in contigs. However, in some cases, it may be necessary to check BEAP singlets for exonic sequence if little sequence homology or no flanking sequence exists in the introns of genes. Template sequence comprised of up to 1–5 Mbs total bases can be used to construct contigs depending on computing power and E-value stringency level desired. Changing the E-value and word size generally resulted in the expected result- more sequences queried with a lower E-value or word size and less sequence queried with a higher E-value or larger word size (see Additional File 1.) Clearly, it is important to consider the specificity and precision of the retrieved gene sequence in choosing a lower or higher E-value in BEAP respectively. Both local and network (more current, web-based) databases can be used to query sequence for assembly with little difference in performance. Repetitive template sequence had a limited effect on BEAP, with some reduction in contig size and number as percentage of repetitive sequence content increased.

### Performance of the BEAP GUI

The BEAP GUI allows for easy identification of sequence mismatch that may represent possible SNPs. In the nucleotide view, an option is present to colour base mismatches between sequence reads. This nucleotide mismatch tool can be turned on or off as desired by the user. A base in the sequence data that does not match the equivalent base of the contig is coloured either red or green when this feature is turned on. A red base means that the base does not match, but the score for that base in the contig is high (> 50). A green base means that the base does not match, and the score for that base in the contig is low (< 50). Identification of sequence variation may indicate the presence of SNPs, poor sequence quality, or an improperly placed sequence within a contig. The ability to code these sequences allows a researcher to assess the quality of the sequence assembly and the potential of polymorphic nucleotide variation. The ability to detect SNPs would have immediate impact as genetic markers to expedite alignment of contigs to genetic maps or for identification of gene duplication. Refinement of this tool is required to add confidence in a single mismatch as a putative SNP.

### Application: assessment of the bovine dwarfism locus assembly

**Figure 1:**
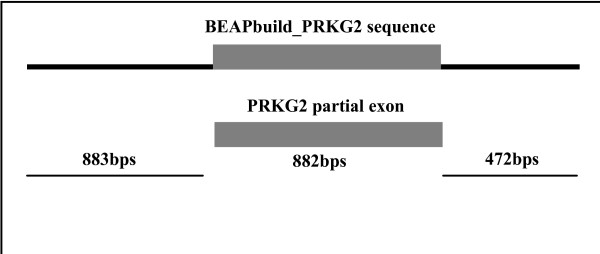
**Demonstration of sequence extension using BEAP**. Example of a contig created in the Angus dwarfism locus by BEAP. The contig is aligned, using the align two sequences tool at NCBI [16], to the first BLAST match to the template sequence retrieved from NCBI. The alignment shows both successful retrieval of target sequence in cattle and nearly tripling in the size of the template for primer design and lab analysis (883 bps for individual sequence vs. 2237 bps for constructed contig). The score, expect, identity and gap statistics show the contig was used in its entirety, with no gaps and 100% sequence match.

Table 1 presents a summary of the genes retrieved, their sizes and a sequence similarity comparison to human. The sequence similarity between human and cattle ranged from 85.3% to 95.1%. An example of assembled sequence is presented in Figure 1 along with an alignment of an individual sequence member to a constructed contig using NCBI's BLAST 2 sequences tool [6] to demonstrate how BEAP extended template sequences. Analysis of the Angus cattle dwarfism locus demonstrates the potential application of BEAP in a low draft sequence. Our initial test using pre-genome assembly sequence from cattle created a large number of contigs, encompassing the vast majority of genic regions and flanking intronic sequence for the dwarfism locus. Twenty genes were identified in cattle based on BLAST results (Figure 1). Use of current sequence draft data for the cow also created a wealth of sequence data for positional cloning of the locus. The ability to use local and network databases shows the flexibility of BEAP for different researchers needs. The sequence data created was used to design primers for positional candidate gene analysis and eventually for the discovery of the causative mutation for Angus dwarfism (Koltes et al., submitted manuscript).

**Table 1:** Bovine genes discovered in the Angus dwarfism locus by BEAP (prior to bovine assembly.)

Gene	Size^1^	% Similarity to Human^2^	% of transcript retrieved^3^
ANTXR2	167.52 (3551)	90.6	29.7
ANXA3	58.69 (1443)	93.3	22.9
BMP2K	102.55 (2158)	91.0	35.5
BMP3	22.61 (1774)	89.4	47.9
COPS4	40.73 (1747)	93.7	100
FGF5	24.33 (5287)	89.8	100
FRAS1	486.29 (15213)	90.8	21.2
GDEP	34.2 (521)	86.0	100
GK2	1.67 (1677)	87.9	100
HEL308	48.50 (3559)	92.9	59.1
HNRPD	20.62 (2200)	95.1	100
HPSE	42.69 (3705)	93.3	66.4
MASA	24.63 (5113)	89.7	9.2
MRPL1	89.95 (1165)	89.9	24.6
PAQR3	21.48 (3990)	92.8	90.7
PLAC8	24.7 (1391)	-	0
PRDM8	24.04 (2536)	91.6	76.5
PRKG2	116.38 (3328)	94.6	100
SCD4	138.88 (1994)	90.1	100
THAP9	19.53 (3627)	90.0	13.8

^1^The genomic sequence, introns and exons, is given in kilobases (kbs) and the (transcript size is presented in parenthesis, in bps). All data was queried in January of 2005, prior to completion of the bovine genome. The size estimates are based on human orthologs as found at UCSC genome browser at

^2^The sequence similarity between human and bovine genes was determined by comparing exonic sequence only.

^3^The percentage of transcript retrieved by BEAP is based on the size of the human transcript.

## Discussion

### BEAP: A foundation for comparative based genomic characterization

A web-based interface is currently being planned to allow wider public access to BEAP. Wider access to BEAP will greatly expedite analysis of draft genomes, which would otherwise require considerable individual effort. To facilitate retrieval of template sequence, we plan to provide links to ENSEMBL, TIGR, NCBI, UCSC, and RH maps relevant to livestock species available at the NAGRP website [7]. Livestock sequence databases are also available through these links. Links to plant databases, including TAIR [8] and plant genome db [9] could also be provided. Additionally, we would like to add a front end template sequence processing component to screen template sequence for repetitive sequence elements facilitated by repeat masker [5]. If desired, users could choose other contig and sequence assembly viewers, e.g [10-12].

Downstream applications of BEAP output would include links to query NCBI, UCSC and ENSEMBL to view map positions and exon/intron boundaries of genes. Alternatively, access to these databases is facilitated by ECR viewer [13], part of the dCode project, which finds evolutionarily conserved sequences and provides graphical sequence analysis using Zpicture, rVista, and Mulan [14]. In addition, if high homology is present between template and query sequence, programs such as Mulan can use comparative genomics to assess the localized quality of sequence assembly using mega dot plots comparing template and queried sequences for limited distances. This software also allows cross-species annotation. The Avid software could serve to order BEAP contigs [15].

During the development of BEAP, a similar tool was published to harness trace sequences for assembly in to contigs [16]. BEAP differs from this software in that it works locally, and therefore more quickly. It also allows users more flexibility in BLAST options including a wider variety of searchable sequence databases. BEAP also facilitates visualization and quality assessment of sequences and detection of possible SNPs.

## Conclusion

Researchers need to have some flexibility to adapt to each new genome depending on genomic complexity and gene content. A variety of options in the BEAP process were designed to help researchers tackle a wide range of challenges. BEAP is not limited to bovine or animal applications. Any sequence database can be queried or used as a template sequence. The user can specify local (megaBLAST) or remote (BLASTn) database querying, stringency of BLAST hits (E-value), and word size within the megaBLAST option. Users have great flexibility in how to use BEAP output. The BEAP package could be very useful to researchers working with draft quality genome sequence.

## Competing interests

The authors declare that they have no competing interests.

## Authors' contributions

Z-LH designed, updated and participated in testing of the software and provided the vision to create the viewer. JEK conceived ideas to build and enhance the software and performed software tests and drafted the manuscript. EF created the BEAP GUI and assisted with writing the manuscript. JMR conceived the idea of the software, assisted in conceptual improvement of the software, as well writing of the manuscript.

## Supplementary Material

Additional file 1 **Additional materials.** This data describes BEAP testing and shows how optimal setting for the BLAST options within BEAP were determined. Click here for file

Additional file 2 **BEAP software.** The BEAP software is included in this file. Click here for file
