# Supplementary material for: BEAP: The BLAST Extension and Alignment Program- a tool for contig construction and analysis of preliminary genome sequence
Source: BMC Res Notes. 2009 Jan 22;2:11. doi: 10.1186/1756-0500-2-11 (PMC2642851; doi:10.1186/1756-0500-2-11)
Supplement: Additional file 1 — Additional materials. This data describes BEAP testing and shows how optimal setting for the BLAST options within BEAP were determined. [file 1756-0500-2-11-S1.doc]

**ADDITIONAL FILE 1**

**Introduction to BEAP rationale**

A variety of computational methods have been developed to analyze and assemble sequences. Perhaps the most commonly used method for sequence comparison is the basic local alignment and search tool (BLAST) [1,2] Other tools such as VISTA [3], and CLUSTALW [4] are also available; however, these tools do not have as many options for adjustment of sequence retrieval or assembly analysis as BLAST.

The BLAST tool can be harnessed to retrieve homologous sequences for assembly. Comparative sequence analysis is a powerful method to identify and assemble genes and regulatory elements [5]. These methods require template genomes, which act as a seed to find and assemble orthologous target genetic sequence for annotation and wet lab experiments. Draft sequences for many species are usually stockpiled in a database and used to build and rebuild contigs and scaffolds a few times per year. Sequences may be available immediately to make a contig, but may not be assembled yet due to logistical constraints (NCBI trace archive statistics at http://www.ncbi.nlm.gov/Traces/trace.cgi).

Raw gene sequences are often short, as seen in shotgun sequence sizes or small contigs at best. Sequence assembly programs, such as CAP3, have been widely used to assemble genomes into contig scaffolds [6]. More recently, new programs such as PCAP and Arachne have been used to assemble genomes with more flexibility [7, 8]. Unfortunately, these programs require more computing power and are difficult to implement compared to CAP3.

We introduce BEAP, the **B**LAST **E**xtension & **A**lignment **P**rogram as a tool to search and assemble localized genomic regions. We designed BEAP to assemble overlapping DNA sequences into contigs using a template genome selected by the user. The functions built into BEAP include: 1) contig assembly within localized chromosome blocks of interest (i.e. a QTL confidence interval); 2) ability to annotate sequences based on comparison to a template genome; 3) cross-species comparative analysis at the gene level, and; 4) visual interface for analysis of sequence features, quality and *in silico* SNP detection. These assembled contigs could be used as template sequence for primer design in the process of fine-mapping and positional cloning in a linkage mapping analysis.

The BEAP tool is one method for querying and extending existing genomic sequence in a genome that is not fully sequenced. BEAP can help researchers find template sequence for positional cloning and discovery of SNP markers. It is vital that good template sequence be defined for the BEAP process and that repetitive sequences are minimized prior to using BEAP. The results presented describe how BEAP was used to create contig sequences in cattle prior to the completion of sequencing. The BEAP GUI allows sequence assembly assessment and detection of possible SNPs. The GUI allows close assessment of the quality of individual sequences and whole contigs prior to use for wet lab work. Output from BEAP can be used to find genes using evolutionary comparisons between a reference genome and a species with incomplete genome sequence, find template sequence for PCR to do positional gene cloning as well for the detection of possible sequence variation. Our results demonstrate that BEAP was capable of producing contigs useful for fine-mapping and candidate gene analysis that lead to the discovery of the Angus dwarfism mutation. Updates to the program will make it more widely accessible on the web and improve BEAP’s ability to construct contigs and perform sequence analysis. The use of BLAST comparisons to other annotated species will allow a new view of genomic features in previously unannotated sequence in real time, expediting the genomic analysis in newly sequenced organisms.

**ADDITIONAL BEAP TESTING DETAILS**

All trial runs of BEAP used the same settings, with the exception of the factor tested. The same template sequence was used for testing in each case, with the exception of tests looking at repetitive sequence content. Tests using increased sequence sizes used the same sequence used in the other trials with additional contiguous sequence when larger sequences were tested. Default test settings used network BLAST to query six bovine databases at NCBI, include: BAC end sequence, trace expressed sequence tagged sites (EST), trace other sequence, trace whole genome sequence (WGS), high throughput genome sequence (HTGS), and EST databases. The E-value was set to e-30 when differences in E-value were not being queried. The database queries using megaBLAST were always the same, and included the *Bos taurus* unique sequence and *Bos taurus* tiger gene databases. In some cases, following initial tests, the E-value was adjusted as needed to attempt to force BEAP to run when no result could be obtained. These changes are noted and reported only when the revised method was successful and the initial test failed in creating contigs.

**Test statistics**

BEAP performance was assessed based on the number and size of contigs created.

**BEAP TESTING RESULTS**

**Influence of repetitive sequence**

To determine the effect of repetitive sequence elements on BEAP’s contig number and size output, we tested how BEAP would handle sequence with and without repeat masking. Each BEAP test was performed with or without repetitive sequence using a 200 kilobases (kbs) region of HSA4 from 13-13.2 Mbs as the template and bovine as the queried sequence. Using Repeat masker to filter repetitive sequences, 14 contigs were constructed ranging in sizes from 493 base pairs (bps) to 2,147 bps with an average size of 1,330 bps. All contigs totaled 18,612 bps. The same analysis without Repeat Masker constructed 15 contigs with nearly identical contig sizes. The total number of bases constructed into contigs was 20,021 bps.

The level of repetitive sequence was tested to determine BEAP’s tolerance level and performance with varying levels of repetitive sequence elements. Repetitive sequence elements were evaluated with Repeat Masker and unmasked sequences with total repetitive sequence ranging from 0% to 50% were used as template for BEAP. All sequences analyzed with BEAP were approximately 20kbs in size. Template sequence with 0% repetitive sequence resulted in 24 contigs with an average size of 1,825 bps ranging in size from 254 bps to 6,117 bps. The total number of bases constructed into contigs was 43,803 bps. With 10% repetitive template sequence, seven contigs were constructed with an average size of 1,152 bps spanning a range of 493 bps to 1,561 bps. The total number of bases constructed into contigs was 8,062 bps. With 50% repetitive template sequence, five contigs were constructed with an average size of 4,246 bps, with contigs ranging in size from 393 bps to 19,001 bps. A total of 21,228 bases were queried and constructed. Results are summarized in **Figure 2**.

**Figure 2A**


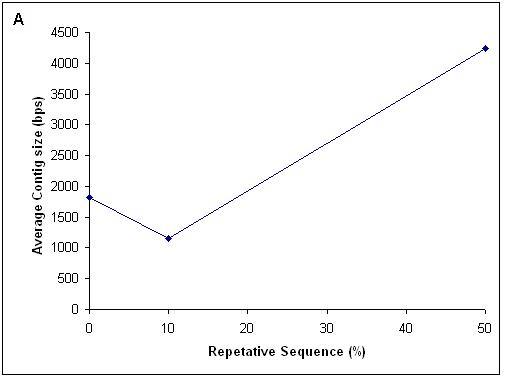


**Figure 2B**


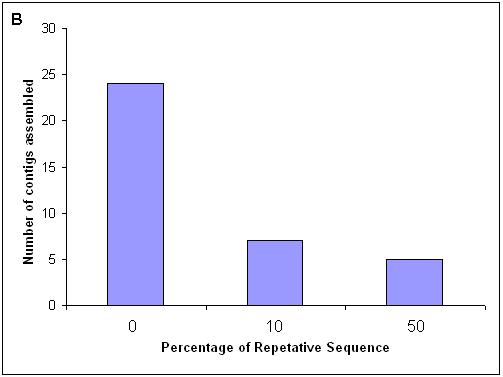


**Figure 2.** The relationship between percentage of repetitive sequence elements in BEAP template and size and number of contigs assembled. A) A non-linear trend in average contig size with increased input of repetitive sequence as template sequence for BEAP. B) Decreased number of contigs produced by BEAP when the percentage of repetitive sequence elements in the template sequence is increased.

**Capacity to handle total template sequence size**

To determine if BEAP could handle blocks of sequence comparable to the size of a QTL confidence interval, total template input size was tested. Human genomic sequence blocks of 50 kbs, 1 Mb and 5 Mbs were used as BEAP template sequence. Each sequentially larger genomic segment contained the same sequence as found in the smaller template file, plus additional sequences to make the larger template size. The 50 kbs template sequence was processed resulting in three contigs with an average size of 5,198 bps and a range of 4,117 bps to 7,342 bps. A total of 15,594 bps were queried and constructed. The 1 Mbs template size resulted in 31 contigs with an average size of 1,750 bps ranging in size from 740 bps to 7,301 bps. A total of 54,338 bps were queried and constructed into contigs. Last, a 5 Mbs segment of template sequence was placed into the BEAP program. Initially, the computer used the maximum amount of memory and was unable to process the data. Additional trials were performed with two different segments of the genome. The amount of unmasked sequence varied greatly; however, BEAP was unable to process the data without exhausting the available memory. In order to construct contigs, the E-value had to be increased and fewer databases (2) were queried using the megaBLAST option instead of network BLAST. Raising the E-value to e-120 and using megaBLAST finally resulted in constructed contigs. There were 18 contigs constructed at an average of 208 bps per contig. A total of 3,747bps were constructed with minimum size of 80 bps and maximum size of 1,000 bps.

**Influence of querying BLAST databases on local server vs. the web**

Computation time is an important factor in the assembly process. To compare contig size and number, megaBLAST and network BLAST options were tested to assess the feasibility of using local vs. web based sequence database querying. Both methods used an E-value of e-30. The megaBLAST option was set to a wordsize of 20. The local BLAST queried two databases, where as the network BLAST queried 6 databases. A total of 22 contigs were constructed using the network BLAST method with an average size of 2,815 bps and a total of 59,117 bps constructed. The maximum contig size was 9,981 bps and the minimum was 493 bps. The local BLAST method resulted in 14 contigs constructed with a total of 42,779 bps. The average contig size was 3,056 bps with a maximum size of 10,325 bps and a minimum size of 383 bps.

**The influence of adjusting network BLAST settings on BEAP contig number and size**

The optimal E-value was tested using E-values ranging from e-30, e-60, to e-120. The same template sequence was used for all tests. The results from e-30 were 33 contigs consisting of a total of 87,453 bps for all contigs. The maximum contig size was 11,225 bps and the minimum was 430 bps. Using e-60 as the threshold resulted in 25 contigs with an average size of 3130 bps and a total size of 78,259 bps summed across all contigs. The maximum contig size was 11,225 bps and the minimum was 753 bps. When the E-value threshold was raised to e-120, 17 contigs were constructed with an average size of 3,979 bps and a total of 67,644 bps for all contigs constructed. The maximum contig size was 11,225 bps and the minimum was 1,239 bps. A summary of results is provided in **Figure 3**.

**Figure 3A**


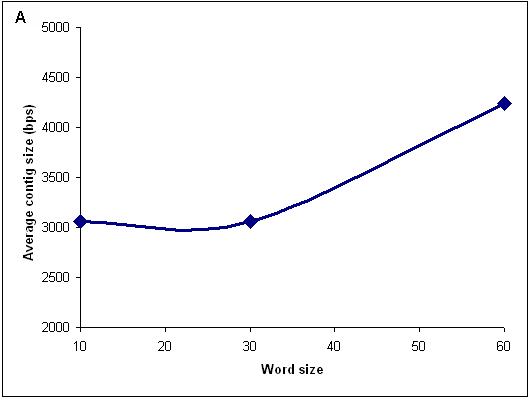


**Figure 3B**


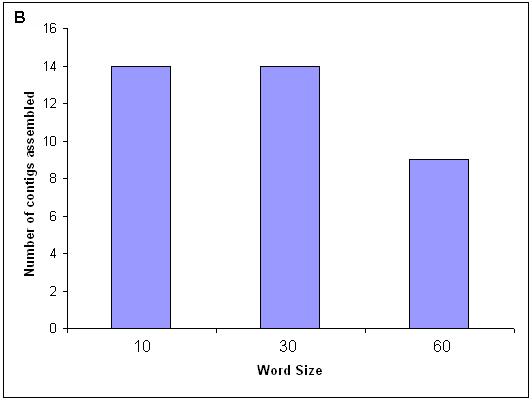


**Figure 3.** The impact of increased E-value stringency on **A**) average contig size, and **B**) number of contigs constructed.

**Impact of adjusting the word size of megaBLAST on contig number and size**

In the megaBLAST option, three different word sizes were tested, 10, 30, and 60. The test using 10 as word size resulted in 14 contigs with an average size of 3,056 bps and a total size of 42,779 bps constructed for all contigs. The maximum contig size was 10,325 bps and the minimum size was 383 bps. Adjusting the word size to 30 resulted in nearly the same results, with only negligible changes in the size of one contig. Changing the word size to 60 resulted in 9 contigs with an average size of 4,243 bps and a total of 38,189 bps across all contigs. The maximum contig size was 10,320 bps and the minimum was 2,089 bps. A summary of the results is provided in **Figure 4.**

**Figure 4A**


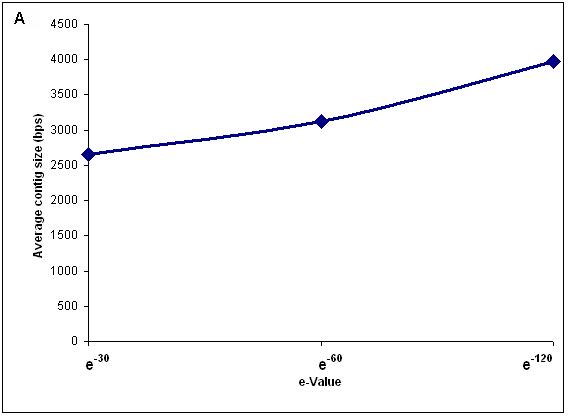


**Figure 4B**


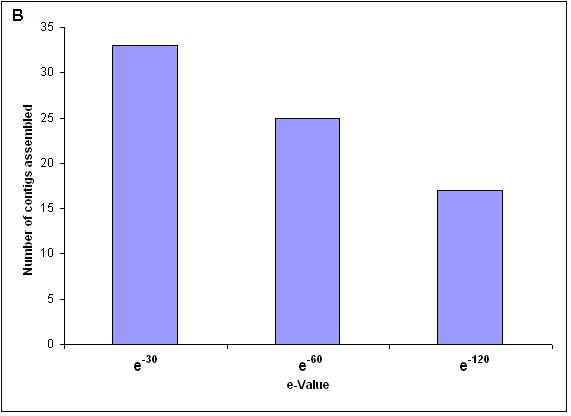


**Figure 4.** The effect of increased word size on A) average contig size, and B) number of contigs constructed.

.

**DISCUSSION**

**Influence of repetitive sequence**-little difference was observed with or without the use of repeat masker on the number and average size of contigs produced by BEAP. It was evident that the increased amount of repetitive sequence within BEAP resulted in fewer contigs and a lower amount of total sequence assembled.

**Capacity to handle total template sequence size**- using the specified parameters of the experiment, 1Mb was the maximum amount of sequence that could be constructed efficiently. Template sizes up to 5Mb could be achieved but only by greatly altering the E-value stringency and limiting the number of databases queried. Greater computational power may allow larger volumes of sequence to be assembled.

**Influence of querying BLAST databases on local server vs. the web**- Little difference was observed between results obtained from local and network database querying. Computational time was the biggest factor, and was greatly reduced using the local database. Fewer, less recently updated databases were queried on the local database, however, the overall assembly appeared to be unaffected by these restraints.

**The influence of adjusting network BLAST settings on BEAP contig number and size**- resulted in reduction in the size and number of contigs constructed as expected. In general, lower E-values resulted in more and larger contigs. Care should be taken to prevent loss of specificity by lowering the E-value to low when genes have paralogues or large conserved genomic sequence domains.

**Impact of adjusting the word size of megaBLAST on contig number and size**- generally larger word sizes resulted in fewer and smaller contigs, as would be expected. Smaller word sizes may allow for better comparison across species by allowing BLAST flexibility to allow gaps and sequence variation.

**SUMMARY OF ALL TESTS**

A summary of tests is presented below in **Table 1**.

**Table 1**

| **Test** | **Simple** | **Intermediate** | **Complex** |
| --- | --- | --- | --- |
| **Genomic content** | Exon only | Exon + UTRs | All Exon & Intron sequence |
| **Input seq. size** | <1000 bps | 1000-5000 bps | >5000 bps |
| **Run time** | 100 input seq. | 500 input seq. | 1000 input seq. |
| **Optimal E-value** | e-30 | e-60 | e-120 |
| **Word size** | 10 | 30 | 60 |
| **# Databases** | 1 | 6 | 12 |
| **# “BEAP” rounds** | 10 | 30 | 60 |
| **Amount repetitive sequence** | 0% | 10% | 50% |
| **Total template input** | 50 kbs | 1 Mbs | 5 Mbs |
| **Repeat masker** | Yes | No |  |
| **BLAST database location** | **Local (megaBLAST)** | **Remote (netBLAST)** |  |

**Table 1.** Tests performed with BEAP under various conditions. Comparisons of megaBLAST vs. netBLAST were performed with the same sequence, but different databases. Default settings for testing were netBLAST using six databases, including: BAC end sequence, expressed sequence tagged site (EST), EST trace, other trace sequence whole genome shotgun sequence trace, and high throughput genome sequence databases and an E-value of 1e-30.

**Figure 5- LOGIC TREE OF BEAP PROCESS**


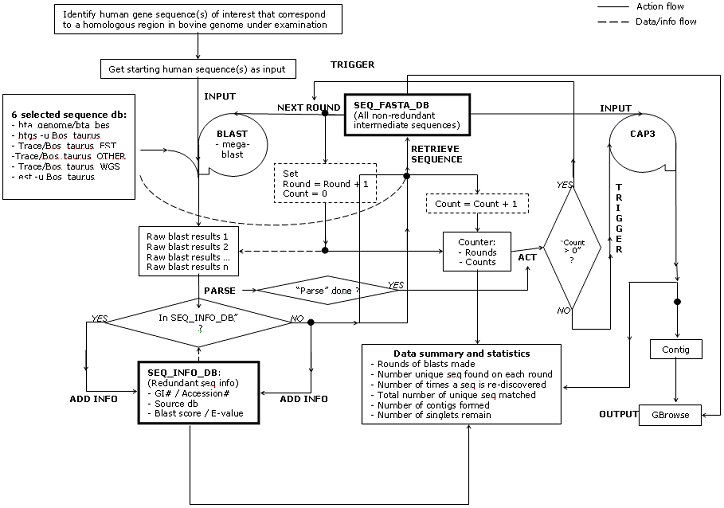


GUI

**Figure 1**. Schematic of the BLAST Extension and Alignment Program (BEAP). A template sequence is used by BLAST to query a species of interest for a localized chromosomal block ≤ 5 Mbs. BEAP performs repetitive BLAST cycles using retrieved sequence as a template until no new sequence is retrieved. Once BLAST is completed, CAP3 assembles the sequence to create contigs for downstream analysis. The process is similar to an *in silico* chromosome walking experiment. Output can be assessed using the BEAP GUI viewer or any software desired by the user.

**REFERENCES**

1. Altschul S, Gish W, Miller W, Myers EW, Lipman DJ: **Basic local alignment search tool.** *J Mol Biol* 1990*,* **215**: 403-410

2. Altschul S, Madden TJ, Schaffer AA, Zhang J, Zhang Z, Miller W, Lipman DJ: **Gapped BLAST and PSI-BLAST: a new generation of protein database search programs.** *Nucleic Acids Res* 1997, **25**:3389-3402

3. Frazer K, Pachter L, Poliakov A, Rubin EM, Dubchak I: **VISTA: computational tools for comparative genomics.** *Nucleic Acids Res* 2004, 32(Web Server issue): W273-279

4. Thompson J, Higgins DG, Gibson TJ: **CLUSTAL W: improving the sensitivity of progressive multiple sequence alignment through sequence weighting, position-specific gap penalties and weight matrix choice.** *Nucleic Acids Res* 1994, **22**: 4673-4680

5. Margulies E, Cooper GM, Asimenos G, Thomas DJ, Dewey CN, Siepel A, Birney E, Keefe D, Schwartz AS, Hou M, Taylor J, Nikolaev S, Montoya-Burgos JI, Löytynoja A, Whelan S, Pardi F, Massingham T, Brown JB, Bickel P, Holmes I, Mullikin JC, Ureta-Vidal A, Paten B, Stone EA, Rosenbloom KR, Kent WJ, Bouffard GG, Guan X, Hansen NF, Idol JR, Maduro VV, Maskeri B, McDowell JC, Park M, Thomas PJ, Young AC, Blakesley RW, Muzny DM, Sodergren E, Wheeler DA, Worley KC, Jiang H, Weinstock GM, Gibbs RA, Graves T, Fulton R, Mardis ER, Wilson RK, Clamp M, Cuff J, Gnerre S, Jaffe DB, Chang JL, Lindblad-Toh K, Lander ES, Hinrichs A, Trumbower H, Clawson H, Zweig A, Kuhn RM, Barber G, Harte R, Karolchik D, Field MA, Moore RA, Matthewson CA, Schein JE, Marra MA, Antonarakis SE, Batzoglou S, Goldman N, Hardison R, Haussler D, Miller W, Pachter L, Green ED, Sidow A.: **Analysis of deep mammalian sequence alignments and constraint predictions for 1% of the human genome.** *Genome Res* 2007, **17**: 760-774

6. Huang X, Madan A: **CAP3: A DNA sequence assembly program.** *Genome Res* 1999, **9**: 868-877

7. Batzoglou S, Jaffe DB, Stanley K, Butler J, Gnerre S, Mauceli E, Berger B, Mesirov JP, Lander ES: **ARACHNE: a whole-genome shotgun assembler.** *Genome Res* 2002, **12**: 177-189

8. Huang X, Wang J, Aluru S, Yang SP, Hillier L: **PCAP: a whole-genome assembly program**. *Genome Res* 2003, **13**: 2164-21
